# Effects of a Sleep Health Education Program for Children and Parents on Child Sleep Duration and Difficulties: A Stepped-Wedge Cluster Randomized Clinical Trial

**DOI:** 10.1001/jamanetworkopen.2022.23692

**Published:** 2022-07-26

**Authors:** Karen Bonuck, Akilah Collins-Anderson, Clyde B. Schechter, Barbara T. Felt, Ronald D. Chervin

**Affiliations:** 1 Montefiore Medical Center, Albert Einstein College of Medicine, Bronx, New York; 2 Brown School (Public Health) at Washington University in St Louis, St Louis, Missouri; 3 Department of Pediatrics, University of Michigan, Ann Arbor; 4 Sleep Disorders Center, University of Michigan, Ann Arbor; 5 Department of Neurology, University of Michigan, Ann Arbor

## Abstract

**Importance:**

Preschool-aged children often lack sufficient sleep and experience sleep difficulties. A consistent bedtime routine, falling asleep alone, and other sleep practices reduce difficulties and increase sleep duration.

**Objective:**

To evaluate the effects of a preschool-based sleep health literacy program on children’s sleep duration and difficulties and on parent sleep knowledge, attitudes, self-efficacy, and beliefs 9 and 12 months after the program.

**Design, Setting, and Participants:**

This stepped-wedge cluster randomized clinical trial was implemented across the 2018-2019 school year. Head Start preschool personnel delivered interventions and collected outcomes data at baseline and 4 follow-ups. Seven Head Start agencies across New York State were randomized to implement interventions in either fall 2018 or winter and spring 2019. Outcomes were ascertained at 9- and 12-month follow-up. From March 19 through September 28, 2018, Head Start staff recruited (a) English- or Spanish-speaking parents (b) of children 3 years of age on or about September 2018 (c) who planned to remain at the site through the school year. Altogether, 519 parent-child (aged 3 years) dyads completed baseline and (any) follow-up data.

**Interventions:**

A 2-week classroom curriculum for children, a 1-hour parent workshop, and 1-on-1 parent discussions at home or school.

**Main Outcomes and Measures:**

Outcomes were the pre- vs postintervention differences measured at baseline and 9-month follow-up for parent-reported child school-night sleep duration per sleep logs, mild or moderate sleep difficulties per a validated questionnaire, and the total and domain scores for parent sleep knowledge, attitudes, self-efficacy, and beliefs. A modified intention-to-treat analysis excluding participants with only baseline data was used.

**Results:**

The mean (SD) age at enrollment of 519 children was 2.7 (0.1) years, 264 (50.9%) were girls, 196 (37.8%) lived in Spanish-speaking households, and 5 (0.9%) identified as Alaskan Native or American Indian, 17 (3.2%) as Asian American or Pacific Islander, 57 (10.8%) as Black, 199 (37.8%) as White, and 63 (12.0%) as other. Mean sleep durations increased nonsignificantly from baseline by 5.6 minutes (95% CI, −2.3 to 13.6 minutes; *P* = .17) at 9-month follow-up and by 6.8 minutes (95% CI, 0.2-13.7 minutes; *P* = .06) at 12-month follow-up. There was a slight improvement in parental knowledge (1.13 unit increase from baseline; 95% CI, 0.13-2.12 units), but no significant outcomes for parent sleep attitudes (0.16 unit increase from baseline; 95% CI, −0.46 to 0.77 units), self-efficacy (−0.13 unit decrease from baseline; 95% CI, −1.02 to 0.76 units) and beliefs (−0.20 unit decrease from baseline; 95% CI, −0.56 to 0.16 units). Intervention effects for child sleep difficulties were not significant (odds ratio, 1.13; 95% CI, 0.62-2.09). Fewer than 1 in 4 parents accurately perceived their child’s sleep difficulty at 12 months.

**Conclusions and Relevance:**

The findings of this large pragmatic, stepped-wedge cluster randomized clinical trial, albeit largely negative, may have implications for the sustained impact, focus, and potential population-level effects of sleep education programs. Future research should evaluate the effects of more recurrent programming that emphasizes recognition of sleep problems and whether small increments of sleep across months and years in early childhood have meaningful effects.

**Trial Registration:**

ClinicalTrials.gov Identifier: NCT03556462

## Key Points

**Question:**

Does a sleep health education program for children and parents in Head Start improve childhood sleep and related outcomes?

**Findings:**

This stepped-wedge cluster randomized clinical trial of 519 parent-child (aged 3 years at enrollment) dyads assessing the effects of a 2-week classroom curriculum for children, 1-hour parent workshop, and 1-on-1 parent discussions at home or school yielded largely negative 9- and 12-month outcomes for children’s sleep duration and difficulties and caregiver sleep, attitudes, self-efficacy and beliefs.

**Meaning:**

These findings, although negative, provide direction for future research on the sustained impact, focus, and potential population-level effects of sleep education programs.

## Teaser

This stepped-wedge cluster randomized clinical trial evaluates the effects of a Head Start sleep health literacy program on sleep duration and difficulties in children 3 years of age and on parents' sleep knowledge, attitudes, self-efficacy, and beliefs at 9 and 12 months after the intervention.

## Introduction

Young children need sufficient, healthy sleep for optimal cognitive,^1,2,3^ social-emotional,^1,3,4,5^ and physical development^2^ and to reduce obesity risk.^6^ At preschool age (3-5 years), 10 to 13 hours of sleep per day inclusive of naps is recommended.^7^ Yet prior to school entry, up to one-third of US children do not sleep that much.^5,8,9^ In addition, 25% to 30% of preschoolers experience difficulties falling and staying asleep, that is, behavioral sleep problems.^10,11^ Yet optimal sleep hygiene practices at this age—including a consistent bedtime and bedtime routine, falling asleep on one’s own, and limiting screen time before bed—are associated with fewer behavioral sleep problems and longer sleep duration.^12,13^ Parent knowledge and attitudes about child sleep are significantly associated with the quality and quantity of sleep for children 1 to 5 years of age.^14,15^

There is growing attention to sleep health in general^16^ and pediatric^17,18,19^ populations. Research in preschool children (approximately 2.5-5 years of age) has prioritized home-based interventions,^20^ or interventions targeting obesity rather than sleep as a primary outcome.^21^ Despite sleep health disparities,^22,23^ population samples have lacked racial, ethnic, and socioeconomic diversity.^24^ Half of the nation’s preschoolers attend early childhood programs,^25^ in which promoting sleep health is endorsed by parents and staff.^18,26,27^ The federal Head Start early childhood program serves approximately 700 000 diverse, lower-income preschoolers and their families. Research in Head Start finds that healthy sleep improves cognitive and social-emotional function,^3^ and a randomized clinical trial (RCT) of parent and classroom education resulted in 30 minutes’ longer sleep duration at the 1-month follow-up.^15^

Despite evidence that sufficient quality sleep promotes development and brain function, Head Start does not routinely promote sleep health literacy. One reason may be lack of adequate evidence that sleep routines can be changed, sleep problems reduced, and parent knowledge about sleep improved. Thus, we conducted a primary prevention trial of sleep health interventions in Head Start. This RCT of multilevel interventions grounded in the social and ecological model^28,29^ examined the following 3 outcomes at the 9- and 12-month follow-up: (1) child sleep duration, (2) child sleep difficulties, and (3) parent knowledge, attitudes, self-efficacy, and beliefs (KASB) regarding children’s sleep.

## Methods

### Setting

Head Start agencies from urban, suburban, and rural areas across New York State were selected based on having implemented low health literacy education programs (eg, oral health, obesity prevention).^30,31,32,33^ This implementation approach from the Health Care Institute at the Anderson School of Management (University of California, Los Angeles) builds Head Start agency capacity to both collect data and implement interventions. The present report follows the Consolidated Standards of Reporting Trials (CONSORT) reporting guideline for RCTs. The Office of Human Research Affairs at Albert Einstein College of Medicine, Bronx, New York, approved this study. Head Start staff obtained informed consent from participants, and the recruiting staff signed and dated consents on participants’ behalf. Participants were not compensated but received a study-branded child-sized blanket. Agencies received funds to host study-related events (eg, refreshments for the parent workshop).

### Design

We conducted a pragmatic, stepped-wedge cluster RCT. (Pragmatic trials evaluate the effectiveness of interventions under actual practice conditions.) The trial protocol and statistical plan are available in Supplement 1. Head Start agencies were randomly assigned to wedge 1 (4 agencies) or wedge 2 (3 agencies). The 23 sites within those agencies collected data at baseline, before and after intervention implementation in wedges 1 and 2 (fall of 2018 and early in 2019, respectively), and at the 1-year follow-up (September 2019) (eFigure in Supplement 2). This design is often used for routine care interventions that have a favorable ratio of benefit to harm.^34^

### Preimplementation Phase

During the 2.5 years of the preimplementation phase, the study team and partners developed and pilot tested intervention materials and built Head Start capacity to enroll participants, deliver interventions, and collect study data. A kickoff retreat in March 2018, just prior to recruitment, was held to review the logistics and materials.

### Materials

The training and curriculum for teachers, children, and parents in the Early Childhood Sleep Education Program (ECSEP) served as core content.^15^ The ECSEP classroom lessons align with preschool curricula; child and parent education fulfills Head Start performance standards.^35,36^ Parent- and child-facing education materials were available in English and Spanish. Details of study materials and how they were used to deliver interventions are shown in Table 1.

**Table 1.** Intervention Descriptions: Content, Delivery, and Materials

Intervention	Content	Delivery	Materials
**ECSEP**			
Parent workshop	Science of sleep Sleep effects on child’s brain and body Hours of sleep needed Bedtime routines (why, “how to”)	Held at sites During parent meeting 1 wk Before class lessons	1-h PowerPoint presentation Parents receive take-home guide
Classroom lessons	Why children need sleep, bedtime routine steps, etc Modalities: songs, storybooks, teddy bears to model bedtime routines	By Head Start teachers Lessons: 8 sessions in 2 weeks, 40 min/d Small- and large-group activity	Child take-home items: Teddy bear (second week) Book (read in class) Toothbrush and toothpaste Sticker chart and stickers Teacher tools: Curriculum guide, lesson plans, 3 classroom teddy bears, 10 enrichment lessons^a^
**Multimedia materials developed during preimplementation**			
Sleep health flip chart (parent 1-on-1 visit with Head Start staff)^b^	Reviews parent workshop content Additional tailored content: FAQs: night waking, cosleeping, etc Red flags: snoring, daytime fatigue	At home or Head Start site During routine 1-on-1 visits Guides staff and parent dialogue Duration: 20-25 min	Spiral bound, desk sized, full color 26 Pages: 13 for parents, 13 for staff English and Spanish
Sleep health brochure	Summarizes flip chart content Includes bedtime dos and don’ts Sleep goals: parents can write 1-3 goals	Parent receives after flip chart sessions	Trifold brochure English and Spanish
Bedtime challenges brochure	Strategies for fighting bedtime, nighttime fears, shared sleep space, etc	Parent receives after flip chart sessions	1 Page, double sided English and Spanish
Sleep health video	Professional production, content mirrors above materials	Shared via agency social media, website, & newsletter	8-min Video distributed as MP4 audio file English and Spanish

Abbreviations: ECSEP, Early Childhood Sleep Education Program; FAQs, frequently asked questions.

^a^ Supplemental lessons for future use; not implemented during this trial.

^b^ One agency delivered the sleep health flip chart in small groups immediately after the 1-hour parent workshop.

### Training

Researchers trained agency staff to enroll families and administer study instruments. Approximately 1 month prior to the implementation of interventions at each site, developers of the ECSEP (Sweet Dreamzzz, now part of Pajama Program) provided half-day training to staff delivering the parent workshop, classroom lessons, and sleep health flip chart.

### Eligibility

From March 19 through September 28, 2018, Head Start staff recruited (a) English- or Spanish-speaking parents (b) of children 3 years of age on or about September 2018 (c) who planned to remain at the site through the 2018 to 2019 school year. Enrolling only children who were 3 years of age (vs 3 and 4 years) was selected to account for developmental changes in sleep and to increase retention at the 12-month follow-up.

### Randomization

The study statistician (C.B.S.) randomized the 7 agencies to either wedge 1 or wedge 2. Wedge 1 was assigned to deliver study interventions in fall 2018 and wedge 2 implemented interventions late winter or early spring 2019.

### Interventions

The study was implemented across the 2018 to 2019 school year. Head Start staff in each wedge received half-day trainings 1 month prior to implementing the ECSEP 1-hour parent workshop, 2-week classroom curriculum, and guided sleep health flip chart discussion (Table 1). Classroom lessons and materials (eg, teddy bear, book) were provided to every classroom in buildings where there were study participants. Parents of all children in classrooms exposed to the intervention were invited to a parent workshop, which may or may not have been in the same building as the classroom. Thus, more parents and children were exposed to ECSEP and related interventions than participated in the study.

### Outcomes and Measures

#### Demographic Characteristics

Race and ethnicity, preferred language (English or Spanish), and child enrollment in special education (Individual Family Service Plan and Individualized Education Plan) data were abstracted from agency records by Head Start staff. Race and ethnicity data were collected because despite sleep health disparities, such data are limited. Head Start data classifies race as Alaskan Native, American Indian, Asian American, Black, Pacific Islander, White, and other. Ethnicity was classified as Hispanic or not. To explore associations between sleep and childhood obesity, we converted height and weight data from physician records that parents submit at enrollment to Head Start to body mass index *z* scores using the US Centers for Disease Control and Prevention growth references for the year 2000.

#### Child Sleep Duration

Child sleep duration was measured from sleep log data for school nights (Sunday through Thursday). At recruitment, agency staff showed parents how to record their child’s bedtimes and wake times, defined as “lights out” and “when the child woke up to start the day,” respectively. Parents completed 7-day paper (not computer) sleep logs at 5 points: 1 week before and 1 week after implementation in wedges 1 and 2, and at the final 1-year follow-up (follow-up 4). Hypothesized differences in sleep duration for intervention vs control periods were (a) longer duration for intervention periods (primary outcome: difference at follow-up 3, which occurred at approximately 9 months), (b) more than a 30-minute difference at follow-up 3, and (c) a 15-minute difference in duration at follow-up 4. Logs with data for at least 4 of 5 school-night sleep durations were considered evaluable and were included in the analysis.

#### Child Sleep Difficulties

Child sleep difficulties were assessed with the Tayside Children’s Sleep Questionnaire (Tayside). This valid and reliable 10-item tool assesses difficulties initiating and maintaining sleep in children 1 to 5 years of age.^37,38^ This low-literacy (≤6th grade) questionnaire has been used in previous research.^39,40,41^ For this RCT, we reduced the recall period from 3 months to 2 months, and we edited the language for clarity (eg, revised double negatives). The first 9 items were each scored from 1 to 4; a total score of 8 or higher of 36 possible indicated mild to moderate difficulties. The 10th item asked caregivers (yes vs no) whether their child had sleep difficulties. Hypothesized differences between intervention vs control periods were (a) lower mean total scores, (b) lower odds of scores being 8 or higher, and (c) fewer parents reporting a sleep problem (yes vs no) in the 10th item on the Tayside questionnaire. Surveys with at least 7 of 9 items scored were included in analyses (secondary outcome).

#### Parent KASB Questionnaire

The KASB questionnaire reflects content from the ECSEP parent workshop regarding child sleep across the named domains. Parents rated agreement with 27 items on a 5-point scale for knowledge (12 items), attitudes (5 items), self-efficacy (8 items), and beliefs (2 items). One multiple-choice item asked how much sleep a preschooler required. In our pilot RCT of the ECSEP,^15^ 11 of the 12 knowledge items split into 3 factors: a 3-item scale about television, a 5-item scale about bedtime routines, and a 3-item scale about activities before bedtime, with Cronbach α values of 0.85, 0.79, and 0.77, respectively. The 5 attitude items had a Cronbach α of 0.91, the 7 self-efficacy items had a Cronbach α of 0.90, and the 2 belief items had a Cronbach α of 0.94.

Hypothesized differences in parents’ KASB for intervention vs control periods were (a) higher KASB total scores and (b) higher scores for each domain; KASB scales with at least 80% nonmissing responses were included in analyses (secondary outcome).

#### Fidelity

Checklists were developed to assess the fidelity of Head Start staff in delivering the ECSEP parent workshop and classroom curriculum. Research team members applied the checklist to in-person and video observations of its parent and classroom education for (a) training and educational materials (availability and appropriate use), (b) procedure (eg, distribution of teddy bears to children and take-home guides to parents), (c) workshop and lesson plans (steps followed), and (d) qualitative comments.

### Statistical Analysis

#### Power

Mean school-night sleep duration at follow-up 3 (at approximately 9 months) was the primary outcome. Clinically significant effects are evident from an additional 30 to 35 minutes of nighttime sleep.^42,43^ With 7 agencies, a sample of 173 provided 90% power to detect a difference as small as 15 minutes between intervention vs control phases (2-tailed *P* < .05). For parent KASB questionnaires, a secondary outcome, a sample of 450 provided more than 97% power to detect a moderate effect size (Cohen *d*, 0.3) for each of its named scales.

#### Analysis

We conducted a modified intention-to-treat analysis that excluded participants with only baseline data. Summary statistics of continuous variables were calculated as means and SDs; frequency distributions (number and percentage) were calculated for discrete variables. To analyze treatment effects, we fitted generalized linear mixed models with the study outcome variables as dependent variables, an indicator for postintervention status, indicators for the study phase (follow-ups 1-4, baseline as the reference category), and covariates to adjust for age, child sex, Hispanic ethnicity, and race. Random intercepts at the agency and participant levels were included. For continuous outcomes (sleep duration, questionnaire scale scores) the identity link and gaussian distribution were used. The coefficient of the postintervention indicator was the estimate of the mean treatment effect and is presented along with its 95% CI. For dichotomous outcomes (positive Tayside questionnaire score, parent-reported sleep problem), the logit link with a Bernoulli distribution was used. In this model, the treatment was the odds ratio (exponentiated coefficient) of the postintervention indicator, presented with its 95% CI. All analyses were conducted with Stata, versions 16.1 and 17 (StataCorp LLC). A 2-sided value of *P* < .05 or a 95% CI excluding 0 was considered statistically significant.

## Results

In total, 551 parent-child dyads provided informed consent. Excluding 4 participants who were later deemed ineligible and 28 participants who provided only baseline data yielded an analytic sample of 519 dyads. Based on evaluable sleep logs, retention was 395 (76.1%) at follow-up 2, 329 (63.3%) at follow-up 3, and 288 (55.5%) at follow-up 4 (Figure). Altogether, 1142 children across 65 classrooms were exposed to the ECSEP interventions.

**Figure.**
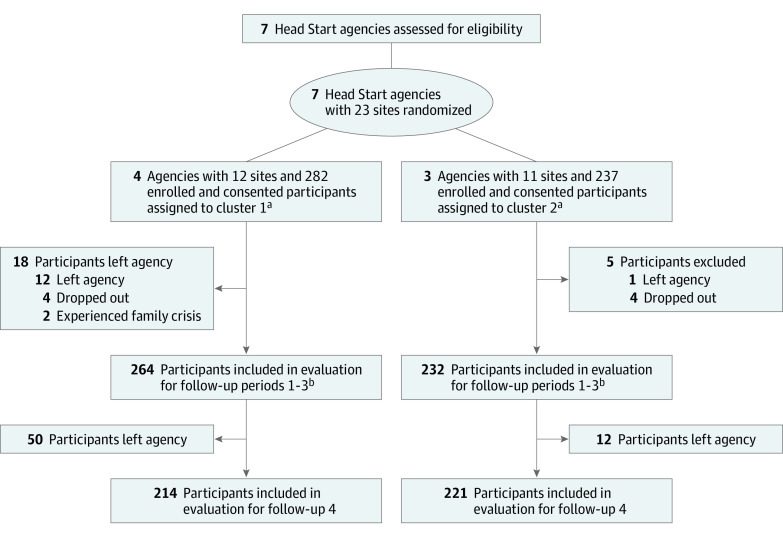
Participant Flowchart ^a^Analytic sample includes participants with at least 1 evaluable study measure (ie, questionnaire or sleep log) at any of follow-up 1, 2, 3, or 4. ^b^Evaluable data were defined as 80% or higher completed data for the sleep log (ie, bedtimes and wake times for 4 of 5 school nights) and the knowledge, attitudes, self-efficacy, and beliefs survey, and 7 of 9 scorable items (78%) for the Tayside Children’s Sleep Questionnaire. Evaluable data at follow-up 2 included 395 logs; at follow-up 3, 329 logs, 332 surveys, and 327 logs plus surveys; and at follow-up 4, 288 logs, 299 surveys, and 282 logs plus surveys.

### Description of Sample at Baseline and End Point Follow-ups

At baseline, of 519 children in the sample, 264 were girls (50.9%), 239 were boys (46.1%), 196 (37.8%) lived in Spanish-speaking households, and 5 (0.9%) identified as Alaskan Native or American Indian, 17 (3.2%) as Asian American or Pacific Islander, 57 (10.8%) as Black, 199 (37.8%) as White, and 63 (12.0%) as other (eTable 1 in Supplement 2).The mean (SD) age of the children at enrollment was 2.7 (0.1) years. Mean body mass index remained within a healthy range throughout the trial^44^ and thus was not included in further analyses.

### Sleep Outcomes by Time Point and Wedge

Across time point and wedge, children averaged at least 10 hours of weeknight sleep (Table 2). From baseline to follow-up 3, the proportion of children with sleep difficulties decreased from 71.3% (201 of 282) to 51.7% (77 of 149) in wedge 1, and from 61.2% (145 of 237) to 43.0% (68 of 158) in wedge 2. Parent-reported (yes vs no) sleep problems were low at baseline and at follow-up 4 in wedge 1 (51 of 281 [18.2%] at baseline; 16 of 142 [11.3%] at follow-up 4) and wedge 2 (13 of 231 [5.6%] at baseline; 9 of 147 [6.1%] at follow-up 4). The KASB total and domain scores appeared to remain stable over time. All remaining results were adjusted for phase (ie, intervention vs control), age, sex, race, ethnicity, and agency.

**Table 2.** Sleep Outcomes by Time Point and Wedge

Instruments & measures	Mean (SD)									
	Wedge 1: 4 agencies (12 sites)^a^					Wedge 2: 3 agencies (11 sites)^b^				
	Control, baseline	Intervention			12-mo Follow-up (follow-up 4)	Control, baseline	Intervention			12-mo Follow-up (follow-up 4)
		Follow-up 1	Follow-up 2	Follow-up 3			Follow-up 1	Follow-up 2	Follow-up 3	
Sleep log										
Sleep duration (Sunday-Thursday), h	10.4 (1.0)	10.5 (0.8)	10.5 (0.8)	10.5 (0.9)	10.3 (0.7)	10.5 (0.9)	10.7 (0.8)	10.9 (0.8)	10.6 (0.7)	10.7 (0.8
Tayside Children’s Sleep Questionnaire										
Total sleep difficulty score	12.0 (6.4)	9.7 (6.1)	NA	9.3 (6.4)	8.6 (6.1)	9.5 (5.1)	9.3 (5.5)	NA	8.7 (4.8)	7.1 (5.2)
Mild to moderate sleep difficulty, % with score ≥8 of 36	71.5	56.3	NA	53.7	53.8	61.9	56.3	NA	59.1	44.2
Parent report of sleep difficulty, % yes (vs no)	18.1	16.9	NA	15.9	11.3	5.6	9.5	NA	5.9	6.1
Knowledge, Attitudes, Self-Efficacy, Beliefs questionnaire										
Total score, range 26-130	102.1 (9.2)	103.4 (9.9)	NA	102.2 (10.1)	102.4 (8.9)	99.8 (11.4)	101.1 (9.2)	NA	101.0 (10.2)	102.0 (11.8)
Knowledge score, range 5-60	37.7 (4.8)	37.3 (5.8)	NA	36.2 (5.1)	35.8 (4.7)	35.6 (4.5)	35.2 (4.2)	NA	34.8 (3.8)	35.1 (5.6)
Attitudes score, range 5-25	21.4 (3.4)	22.0 (3.2)	NA	22.2 (3.3)	22.0 (3.5)	21.6 (3.4)	22.0 (3.1)	NA	22.1 (3.7)	22.3 (3.4)
Self-efficacy score, range 5-40	34.3 (4.5)	35.1 (4.2)	NA	34.9 (4.9)	35.5 (3.9)	34.2 (5.5)	34.9 (4.1)	NA	34.6 (5.3)	35.6 (4.9)
Beliefs score, range 5-10	8.8 (1.8)	9.0 (1.6)	NA	9. 0 (1.5)	9.0 (1.5)	8.6 (1.8)	8.9 (1.4)	NA	8.8 (1.7)	9.0 (1.6)

Abbreviation: NA, not applicable.

^a^ Four agencies: 2 urban, 1 suburban or rural (migrant or seasonal), 1 rural.

^b^ Three agencies: 2 urban, 1 suburban or urban.

### Sleep Duration

The intervention was associated with a nonsignificant mean increase of 5.6 minutes (95% CI, −2.3 to 13.6 minutes; *P* = .17) at follow-up 3 (the primary outcome), and 6.8 minutes (95% CI, 0.2-13.7 minutes; *P* = .06) at follow-up 4 (Table 3). Hispanic ethnicity was associated with less sleep at follow-up 3 (mean decrease, −33.8 minutes; 95% CI, −45.7 to −21.9 minutes) and at follow-up 4 (mean decrease, −32.4 minutes; 95% CI, −43.9 to −20.9 minutes). The time effects (ie, follow-up 1 minus follow-up 4) factored out the phase effects (ie, intervention). Moreover, an effect modification analysis showed similar non-significant effects among children whose baseline school-night sleep duration was less than 10 hours at follow-up 3 (intervention effect, 4.9-minute increase, 95% CI, −9.6 to 19.5 minutes) or follow-up 4 (intervention effect, 8.7-minute increase, 95% CI, −5.1 to 22.4 minutes). In additional analyses with all logs, not just logs with at least 4 of 5 weeknights, the intervention was similarly associated with nonsignificant mean increases of 5.1 minutes at follow-up 3 (95% CI, −2.7 to 13.0 minutes; *P* = .20) and 6.4 minutes at follow-up 4 (95% CI, −0.5 to 13.2 minutes; *P* = .07).

**Table 3.** Sleep Duration Outcomes: Regression Model Effects, Primary Outcomes

Only for logs ≥4 nights	Follow-up 3	*P* value	Follow-up 4	*P* value
	Minutes (95% CI)		Minutes (95% CI)	
Phase				
Intervention	5.6 (−2.3 to 13.5)	.17	6.8 (−0.2 to 13.7)	.06
Time				
Follow-up 1	−0.3 (−7.6 to 7.1)		−1.1 (−7.8 to 5.7)	
Follow-up 2	4.2 (−3.1 to 11.6)		3.4 (−3.3 to 10.1)	
Follow-up 3	2.9 (−6.3 to 12.1)		1.6 (−6.8 to 10.0)	
Follow-up 4	NA		−4.6 (−14.0 to 4.8)	
Age, y	0.6 (−11.7 to 13.0)		−0.9 (−12.7 to 11.0)	
Female sex	1.6 (−5.3 to 8.4)		1.0 (−5.5 to 7.6)	
Hispanic ethnicity	−33.8 (−45.7 to −21.9)		−32.4 (−43.9 to −21.0)	
Race				
Alaskan Native or American Indian	−32.1 (−63.5 to −0.7)		−29.3 (−59.3 to 0.7)	
Asian American or Pacific Islander	−18.7 (−40.7 to 3.2)		−22.3 (−43.4 to −1.2)	
Black	−13.3 (−28.6 to 2.0)		−11.4 (−26.2 to 3.4)	
White	13.5 (−1.7 to 28.7)		12.1 (−2.7 to 26.9)	
Other	7.3 (−7.2 to 21.8)		7.1 (−7.0 to 21.2)	

Abbreviation: NA, not applicable.

### Parental KASB Scores

At follow-up 4, the intervention was associated with a 1.13 unit increase from baseline in knowledge (95% CI, 0.13-2.12 units) (Table 4). There were no significant differences in either total scores (0.81 unit; 95% CI, −1.29 to 2.90 units; *P* = .45) or other domain scores (attitudes: 0.16 unit [ 95% CI, −0.46 to 0.77 units], *P* = .62; self-efficacy: −0.13 unit [95% CI, −1.02 to 0.76] units, *P* = .78; and beliefs: −0.20 unit [95% CI, −0.56 to 0.16 units], *P* = .28). Hispanic ethnicity was associated with lower total KASB score (mean correct, −2.49; 95% CI, −4.78 to −0.20) and knowledge (mean correct, −2.25; 95% CI, −3.37 to −1.13) scores, whereas White (mean correct, −1.20; 95% CI, −2.36 to −0.04) and other race (mean correct, −1.15; 95% CI, −2.23 to −0.07) were associated with lower self-efficacy scores at baseline (eTable 2 in Supplement 2).

**Table 4.** Parent KASB and Child Sleep Difficulties at Follow-up 4: Regression Model Effects, Secondary Outcomes

Model	Item	*P* value
Parent KASB score, mean No. correct (95% CI)		
Total KASB score (higher scores are better)	0.81 (−1.29 to 2.90)	.45
Knowledge	1.13 (0.13 to 2.12)	.03
Attitudes	0.16 (−0.46 to 0.77)	.62
Self-efficacy	−0.13 (−1.02 to 0.76)	.78
Beliefs	−0.20 (−0.56 to 0.16)	.28
Child sleep difficulties; follow-up 4 for all logs, effect (95% CI)		
Total score (higher is worse)	−0.79 (−1.76 to 0.18)	.11
Sleep difficulty, OR (95% CI)^a^	1.13 (0.62 to 2.09)	.69
Sleep problem, OR (95% CI)^b^	0.91 (0.34 to 2.45)	.86

Abbreviations: KASB, knowledge, attitudes, self-efficacy, and behavior; OR, odds ratio.

^a^ Odds of scoring 8 or higher (out of 36), indicating possible to mild sleep difficulty.

^b^ Odds of parent reporting “yes” regarding child sleep problem.

### Sleep Difficulties and Additional Sleep Duration Analyses

At follow-up 4, there were no significant changes in mean Tayside questionnaire total score (−0.79; 95% CI: −1.76 to 0.18), sleep difficulty (odds ratio, 1.13; 95% CI, 0.62-2.09) or parent-reported sleep problem (odds ratio, 0.91; 95% CI, 0.34-2.45) (Table 4).

We explored adjustment for additional site- and participant-level effects for sleep duration across follow-up 3 and follow-up 4 (eTable 3 in Supplement 2). We included an indicator for each of the 23 sites (in addition to random effects for 7 agencies), as well as person-level covariates (eg, language, ethnicity, and race), and site setting as urban vs rural per US Census data. At the participant level, we evaluated effects of participant language (English vs Spanish), Tayside questionnaire total and sleep difficulty scores at baseline, and total Tayside questionnaire score at the observation point. None of these adjustments led to a different estimate of the intervention effect. The adjusted mean sleep duration at baseline was 25 minutes (95% CI, 0.0-51.6 minutes) shorter in the rural site than in urban sites. However, because this finding was based on a single site, this result may not generalize.

### Missing Data

Hispanic ethnicity was missing for 60 participants, and sex was missing for 10 participants. Sleep duration was missing for 75 children at baseline, 70 at follow-up 1, 102 at follow-up 2, and 165 at follow-up 3. We performed multiple imputation by chained equations, with 75 replicates, and applied the Rubin rules, obtaining an intervention effect estimate of 7.8 minutes (95% CI, 0.2-15.4 minutes). In addition, because the data may well be missing not at random, we performed a best case sensitivity analysis in which preintervention missing sleep durations were set to the child’s shortest observed, and postintervention missing sleep durations were set to the longest observed. Missing values of Hispanic ethnicity were set to non-Hispanic, and missing values of sex to female because these categories were more favorable in the observed data. Under these optimistic imputations, the estimated intervention effect was 10.0 minutes (95% CI, 4.7-15.4 minutes).

### Fidelity

In-person observations of parent workshops from 3 agencies yielded positive assessment of staff preparation and thoroughness. On the basis of classroom observations (5 in-person and 1 video) from 6 agencies, teaching teams ably integrated material into the curriculum. Implementation of steps in specific days’ lessons were quantified at 58%, 53%, 33%, 11%, and 50%. In contrast, the evaluator’s report noted that learning objectives for lessons were more consistently met and that “teaching teams in most sites seemed to easily integrate these materials into their ongoing curriculum.”

### Evaluation

Head Start classroom educators rated the ECSEP program and related training on a scale of 1 to 5 (with 5 being best). Across the 7 agencies, results were mean (SD) ratings of 4.82 (0.15) for learning objectives, 4.86 (0.00) for instructors, and 4.80 (0.09) for teaching methods and program content.

## Discussion

This sizeable prospective stepped-wedge cluster RCT implemented a sleep health literacy program for children and parents in Head Start. Assessment of child sleep duration, child sleep difficulties, and parent KASB score outcomes at 9 and 12 months of follow-up failed to show clinically meaningful effects at the individual level. The primary outcome, nighttime sleep duration, increased to clinically insignificant means of 5.6 minutes at 9 months (primary outcome) and 6.8 minutes at 12 months. The intervention led to a slight improvement in parental knowledge, but not in attitudes, self-efficacy, or beliefs. Although child sleep difficulties decreased over time, this decrease was independent of the intervention, perhaps attributable to age or attrition effects. The findings remained essentially unchanged when analyses were adjusted for several covariates (eTable 3 in Supplement 2). Moreover, an effect modification analysis showed a similarly small effect among children whose baseline school-night sleep duration was less than 10 hours. Findings of this trial have implications for the content focus and sustained impact of sleep education programs and potential for population- vs individual-level impacts.

Child sleep difficulties were prevalent: two-thirds of children met criteria for sleep difficulties at baseline and nearly half met the criteria 1 year later. However, few parents thought their child had a sleep problem even after exposure to interventions (weighted average of wedge 1 and wedge 2, 12.5% at baseline and 8.7% at 12-month follow-up). This gap between parent perceptions and measures of child sleep problems is consistent with prior research.^13,18,45^ A review of parent knowledge concluded that “more effort . . . be made to ensure that parents understand children’s sleep requirements, what represents good sleep hygiene and also signs of sleep problems.”^13^ Future parent education may require both reinforcement over time and sharper focus regarding what constitutes a sleep problem.

There is limited translational research on early childhood sleep health or sleep problems in nonclinical sample populations. A previous trial in Head Start of the ECSEP (alone) conducted by our research team found 30-minute increased sleep duration, but no KASB changes, after 1 month.^15^ The shorter follow-up period in that trial, and that developers of the ECSEP delivered the intervention, may underlie the difference in results. In another trial, school nurses delivered sleep hygiene education, including behavioral sleep strategies, to parents of children 5 years of age with behavioral sleep problems. The intervention group experienced fewer sleep difficulties compared with controls at 6 months (standardized effect size, −0.2; 95% CI, −0.4 to −0.04) but not at 12 months (standardized effect size, −0.9; 95% CI, −2.2 to 0.4). Similarly, school-night sleep duration was longer in their intervention group at 6 months (mean difference, 10.9 minutes; 95% CI, 3.4-18.5 minutes) but not at 12 months (mean difference, −0.8 minutes; 95% CI, −0.83 to 6.8 minutes).^46^ Thus, research suggests that sleep education programs will require reinforcement over time along with serial monitoring of parent (eg, KASB) and child (eg, Tayside questionnaire, sleep logs) outcomes.

Small increments in sleep duration during early childhood may have population level effects because sleep affects the developing brain.^47,48^ Our hypotheses of increased sleep duration of 30 minutes after 9 months (follow-up 3) and of 15 minutes after 1 year (follow-up 4) came from studies finding that 30 to 35 minutes’ extra sleep conferred attention and emotional benefits^42^ and neurobehavioral gains equivalent to 2 developmental years.^43^ Those were small studies (<80 participants) of typically developing school-aged children (7-12 years of age) whose sleep was experimentally manipulated across 3 to 5 nights. In real-world conditions, 5 to 7 minutes’ longer nightly sleep across months or years in early childhood may prove meaningful, particularly because nighttime sleep normatively decreases 5 minutes per year between ages 2 and 6 years.^49^ Our precision estimates ranged from 2 minutes’ less nighttime sleep to nearly 14 minutes’ more nighttime sleep. Effects might be amplified in Head Start given the racial and economic disparities in sleep health^23,50^ and the high rates of sleep problems in children with disabilities,^51,52^ who comprise 10% of children in Head Start.^53^

### Strengths and Limitations

This study has strengths, including a large sample size, multiple sites, repeated measurements, a racially and ethnically diverse population, and 1 year of follow-up. Intervention materials were either previously tested (ie, the ECSEP) or collaboratively developed, thus increasing acceptability. Most important, this pragmatic trial evaluated the effectiveness of interventions under real-world conditions.^54^ Site staff delivered interventions that met curriculum goals and during the individual and monthly group parent meetings required by Head Start. In addition, fidelity data were consistent with the pragmatic trial.

The limitations of this study included those associated with stepped-wedge trials, such as respondent burden and practice effects of repeated measures.^55^ The study also lacked objective estimates of sleep duration (eg, actigraphy) although the baseline sleep duration (approximately 10.4 hours) matched that reported by nearly 3000 parents of children aged 3 and 4 years in Head Start.^3^ Additional more systematic fidelity data would provide further context for the interpretation of results.

## Conclusions

The findings from this large, stepped-wedge cluster randomized clinical trial—although largely negative—have implications for the sustained impact, focus, and potential population-level effects of sleep education programs. Sleep education may need to become part of recurrent health-promotion efforts starting in early childhood and continuing through high school. Regarding Head Start specifically, this would mean promoting sleep health similarly to how oral health is promoted, which involves, for example, regular toothbrushing lessons for children. To narrow the gap between parent perceptions and scored ratings of children’s sleep difficulties, the recognition of sleep problems should be central. Finally, data are needed to better understand whether and how small increments of sleep across months and years in early childhood affect development.

## Notes

Supplement 1.
Trial Protocol and Statistical Analysis Plan

Supplement 2. **eFigure.** Stepped-Wedge Cluster Design **eTable 1.** Description of Sample at Baseline and End Point Follow-ups **eTable 2.** Parent Knowledge, Attitudes, Self-efficacy, and Behaviors (KASB): Regression Model Effects **eTable 3.** Additional Analyses: Intervention Effect on Sleep Duration

Supplement 3.
Data Sharing Statement

## References

[1] Reynaud E, Vecchierini MF, Heude B, Charles MA, Plancoulaine S. Sleep and its relation to cognition and behaviour in preschool-aged children of the general population: a systematic review. J Sleep Res. 2018;27(3):e12636. doi:10.1111/jsr.12636 29164715

[2] Chaput JP, Gray CE, Poitras VJ, . Systematic review of the relationships between sleep duration and health indicators in the early years (0-4 years). BMC Public Health. 2017;17(suppl 5):855. doi:10.1186/s12889-017-4850-2 29219078 PMC5773910

[3] Schlieber M, Han J. The sleeping patterns of Head Start children and the influence on developmental outcomes. Child Care Health Dev. 2018;44(3):462-469. doi:10.1111/cch.12522 28891117

[4] Zheng M, Rangan A, Olsen NJ, Heitmann BL. Longitudinal association of nighttime sleep duration with emotional and behavioral problems in early childhood: results from the Danish Healthy Start Study. Sleep. 2021;44(1):zsaa138. doi:10.1093/sleep/zsaa138 32691048

[5] Scharf RJ, Demmer RT, Silver EJ, Stein RE. Nighttime sleep duration and externalizing behaviors of preschool children. J Dev Behav Pediatr. 2013;34(6):384-391. doi:10.1097/DBP.0b013e31829a7a0d 23838583

[6] Miller MA, Bates S, Ji C, Cappuccio FP. Systematic review and meta-analyses of the relationship between short sleep and incidence of obesity and effectiveness of sleep interventions on weight gain in preschool children. Obes Rev. 2021;22(2):e13113. doi:10.1111/obr.13113 33237635

[7] Paruthi S, Brooks LJ, D’Ambrosio C, . Recommended amount of sleep for pediatric populations: a consensus statement of the American Academy of Sleep Medicine. J Clin Sleep Med. 2016;12(6):785-786. doi:10.5664/jcsm.5866 27250809 PMC4877308

[8] Child and Adolescent Health Measurement Initiative. 2018-2019 National Survey of Children’s Health: Indicator 6.25: During the past week, how many hours of sleep did this child get during an average day (count both nighttime sleep and naps) (age 4 months-5 years)/on most weeknights (6-17 years), age 4 months-17 years? 2018. Accessed July 30, 2021. https://www.childhealthdata.org/browse/survey/results?q=7946&g=788&a=13878&r=1

[9] Williamson AA, Mindell JA. Cumulative socio-demographic risk factors and sleep outcomes in early childhood. Sleep. 2020;43(3):zsz233. doi:10.1093/sleep/zsz233 31555826 PMC7315767

[10] Owens JA, Mindell JA. Pediatric insomnia. Pediatr Clin North Am. 2011;58(3):555-569. doi:10.1016/j.pcl.2011.03.011 21600342

[11] Moore M, Bonuck K. Comorbid symptoms of sleep-disordered breathing and behavioral sleep problems from 18-57 months of age: a population-based study. Behav Sleep Med. 2013;11(3):222-230. doi:10.1080/15402002.2012.666219 23205586

[12] Hall WA, Nethery E. What does sleep hygiene have to offer children’s sleep problems? Paediatr Respir Rev. 2019;31:64-74. doi:10.1016/j.prrv.2018.10.005 31076381

[13] McDowall PS, Galland BC, Campbell AJ, Elder DE. Parent knowledge of children’s sleep: a systematic review. Sleep Med Rev. 2017;31:39-47. doi:10.1016/j.smrv.2016.01.002 26899741

[14] Daniel LC, Childress JL, Flannery JL, . Identifying modifiable factors linking parenting and sleep in racial/ethnic minority children. J Pediatr Psychol. 2020;45(8):867-876. doi:10.1093/jpepsy/jsaa034 32447371 PMC7438962

[15] Wilson KE, Miller AL, Bonuck K, Lumeng JC, Chervin RD. Evaluation of a sleep education program for low-income preschool children and their families. Sleep. 2014;37(6):1117-1125. doi:10.5665/sleep.3774 24882907 PMC4015386

[16] Buysse DJ. Sleep health: can we define it? does it matter? Sleep. 2014;37(1):9-17. doi:10.5665/sleep.3298 24470692 PMC3902880

[17] Chaput JP. The integration of pediatric sleep health into public health in Canada. Sleep Med. 2019;56:4-8. doi:10.1016/j.sleep.2018.06.009 30076116

[18] Bonuck KA, Schwartz B, Schechter C. Sleep health literacy in Head Start families and staff: exploratory study of knowledge, motivation, and competencies to promote healthy sleep. *Sleep Health*. 2016;2(1):19-24. doi:10.1016/j.sleh.2015.12.002 PMC4879821 27239486

[19] Meltzer LJ, Williamson AA, Mindell JA. Pediatric sleep health: it matters, and so does how we define it. Sleep Med Rev. 2021;57:101425. doi:10.1016/j.smrv.2021.101425 33601324 PMC9067252

[20] Tinker EC, Garrison MM, Ward TM. Development of the Sleep Health in Preschoolers (SHIP) intervention: integrating a theoretical framework for a family-centered intervention to promote healthy sleep. Fam Syst Health. 2020;38(4):406-417. doi:10.1037/fsh0000546 33591782

[21] Agaronov A, Ash T, Sepulveda M, Taveras EM, Davison KK. Inclusion of sleep promotion in family-based interventions to prevent childhood obesity. Child Obes. 2018;14(8):485-500. doi:10.1089/chi.2017.0235 30109955 PMC6422003

[22] Graham C, Reither EN, Ciciurkaite G, Dev DA, Fargo J. Does context matter? a multilevel analysis of neighborhood disadvantage and children’s sleep health. Sleep Health. 2020;6(5):578-586. doi:10.1016/j.sleh.2020.05.002 32546433

[23] Smith JP, Hardy ST, Hale LE, Gazmararian JA. Racial disparities and sleep among preschool aged children: a systematic review. Sleep Health. 2019;5(1):49-57. doi:10.1016/j.sleh.2018.09.010 30670165 PMC6519123

[24] Schwichtenberg AJ, Abel EA, Keys E, Honaker SM. Diversity in pediatric behavioral sleep intervention studies. Sleep Med Rev. 2019;47:103-111. doi:10.1016/j.smrv.2019.07.004 31450118

[25] US Department of Education National Center for Educational Statistics. Table 202.20: percentage of 3-, 4-, and 5-year-old children enrolled in preprimary programs, by level of program, attendance status, and selected child and family characteristics: 2016. Published 2018. Accessed June 16, 2022. https://nces.ed.gov/programs/digest/d17/tables/dt17_202.20.asp

[26] Bonuck K, Collins-Anderson A, Ashkinaze J, Karasz A, Schwartz A. Environmental scan of sleep health in early childhood programs. Behav Sleep Med. 2020;18(5):598-610. doi:10.1080/15402002.2019.1640222 31318273 PMC6980449

[27] Sadler LS, Banasiak N, Canapari C, . Perspectives on sleep from multiethnic community parents, pediatric providers, and childcare providers. J Dev Behav Pediatr. 2020;41(7):540-549. doi:10.1097/DBP.0000000000000799 32282623 PMC7483133

[28] Bronfenbrenner U. Toward an experimental ecology of human development. Am Psychol. 1977;32(7):513-531. doi:10.1037/0003-066X.32.7.513

[29] Stokols D. Establishing and maintaining healthy environments: toward a social ecology of health promotion. Am Psychol. 1992;47(1):6-22. doi:10.1037/0003-066X.47.1.6 1539925

[30] University of California Los Angeles Anderson School of Management. UCLA Health Care Institute. 2022. Accessed April 20, 2021. https://www.anderson.ucla.edu/centers/price-center-for-entrepreneurship-and-innovation/for-professionals/ucla-health-care-institute

[31] Herman A, Jackson P. Empowering low-income parents with skills to reduce excess pediatric emergency room and clinic visits through a tailored low literacy training intervention. J Health Commun. 2010;15(8):895-910. doi:10.1080/10810730.2010.522228 21170790

[32] Herman A, Nelson BB, Teutsch C, Chung PJ. “Eat Healthy, Stay Active!”: a coordinated intervention to improve nutrition and physical activity among Head Start parents, staff, and children. Am J Health Promot. 2012;27(1):e27-e36. doi:10.4278/ajhp.110412-QUAN-157 22950932

[33] Herman A, Nelson BB, Teutsch C, Chung PJ. A structured management approach to implementation of health promotion interventions in Head Start. Prev Chronic Dis. 2013;10:E155. doi:10.5888/pcd10.130015 24028835 PMC3775358

[34] Mdege ND, Man MS, Taylor Nee Brown CA, Torgerson DJ. Systematic review of stepped wedge cluster randomized trials shows that design is particularly used to evaluate interventions during routine implementation. J Clin Epidemiol. 2011;64(9):936-948. doi:10.1016/j.jclinepi.2010.12.003 21411284

[35] National Resource Center for Health and Safety in Child Care and Early Education. Caring for our children: national health and safety performance standards. 2022. Accessed June 16, 2022. https://nrckids.org/CFOC

[36] Head Start Early Childhood Learning & Knowldege Center. 1302.51 Parent activities to promote child learning and development. 2021. Accessed June 16, 2022. https://eclkc.ohs.acf.hhs.gov/policy/45-cfr-chap-xiii/1302-51-parent-activities-promote-child-learning-development

[37] McGreavey JA, Donnan PT, Pagliari HC, Sullivan FM. The Tayside Children’s Sleep Questionnaire: a simple tool to evaluate sleep problems in young children. Child Care Health Dev. 2005;31(5):539-544. doi:10.1111/j.1365-2214.2005.00548.x 16101649

[38] Spruyt K, Gozal D. Pediatric sleep questionnaires as diagnostic or epidemiological tools: a review of currently available instruments. Sleep Med Rev. 2011;15(1):19-32. doi:10.1016/j.smrv.2010.07.005 20934896 PMC3088759

[39] Corkum PV, Reid GJ, Hall WA, . Evaluation of an internet-based behavioral intervention to improve psychosocial health outcomes in children with insomnia (better nights, better days): protocol for a randomized controlled trial. JMIR Res Protoc. 2018;7(3):e76. doi:10.2196/resprot.8348 29581089 PMC5891669

[40] Novotny R, Davis J, Butel J, . Effect of the children’s healthy living program on young child overweight, obesity, and acanthosis nigricans in the US-affiliated Pacific region: a randomized clinical trial. JAMA Netw Open. 2018;1(6):e183896. doi:10.1001/jamanetworkopen.2018.3896 30646266 PMC6324447

[41] Wilken LR, Novotny R, Fialkowski MK, . Children’s Healthy Living (CHL) Program for remote underserved minority populations in the Pacific region: rationale and design of a community randomized trial to prevent early childhood obesity. BMC Public Health. 2013;13:944. doi:10.1186/1471-2458-13-944 24107083 PMC3851862

[42] Gruber R, Cassoff J, Frenette S, Wiebe S, Carrier J. Impact of sleep extension and restriction on children’s emotional lability and impulsivity. Pediatrics. 2012;130(5):e1155-e1161. doi:10.1542/peds.2012-0564 23071214

[43] Sadeh A, Gruber R, Raviv A. The effects of sleep restriction and extension on school-age children: what a difference an hour makes. Child Dev. 2003;74(2):444-455. doi:10.1111/1467-8624.7402008 12705565

[44] Centers for Disease Control and Prevention, National Center for Health Statistics. CDC growth charts. Reviewed December 7, 2016. Accessed June 27, 2022. https://www.cdc.gov/growthcharts/cdc_charts.htm

[45] Dai Y, Liu J. Parental perceived child sleep problems: a concept analysis. J Spec Pediatr Nurs. 2021;26(2):e12327. doi:10.1111/jspn.12327 33493387

[46] Hiscock H, Quach J, Paton K, . Impact of a behavioral sleep intervention on new school entrants’ social emotional functioning and sleep: a translational randomized trial. Behav Sleep Med. 2019;17(6):698-712. doi:10.1080/15402002.2018.1469493 29757013

[47] Jiang F. Sleep and early brain development. Ann Nutr Metab. 2019;75(suppl 1):44-54. doi:10.1159/000508055 32564032

[48] Kocevska D, Muetzel RL, Luik AI, . The developmental course of sleep disturbances across childhood relates to brain morphology at age 7: The Generation R Study. Sleep. 2017;40(1). 28364462 10.1093/sleep/zsw022

[49] Galland BC, Short MA, Terrill P, . Establishing normal values for pediatric nighttime sleep measured by actigraphy: a systematic review and meta-analysis. Sleep. 2018;41(4). doi:10.1093/sleep/zsy017 29590464

[50] Billings ME, Cohen RT, Baldwin CM, . Disparities in sleep health and potential intervention models: a focused review. Chest. 2021;159(3):1232-1240. doi:10.1016/j.chest.2020.09.249 33007324 PMC7525655

[51] Reynolds AM, Soke GN, Sabourin KR, . Sleep problems in 2- to 5-year-olds with autism spectrum disorder and other developmental delays. Pediatrics. 2019;143(3):e20180492. doi:10.1542/peds.2018-0492 30745433 PMC6398427

[52] Bonuck K, Grant R. Sleep problems and early developmental delay: implications for early intervention programs. Intellect Dev Disabil. 2012;50(1):41-52. doi:10.1352/1934-9556-50.1.41 22316225

[53] Head Start Early Childhood Learning and Knowledge Center. Inclusion of children with disabilities ACF-IM-HS-20-01. 2020. Accessed June 16, 2022. https://eclkc.ohs.acf.hhs.gov/policy/im/acf-im-hs-20-01

[54] Patsopoulos NA. A pragmatic view on pragmatic trials. Dialogues Clin Neurosci. 2011;13(2):217-224. doi:10.31887/DCNS.2011.13.2/npatsopoulos 21842619 PMC3181997

[55] Hemming K, Taljaard M. Reflection on modern methods: when is a stepped-wedge cluster randomized trial a good study design choice? Int J Epidemiol. 2020;49(3):1043-1052. doi:10.1093/ije/dyaa077 32386407 PMC7394949

