# Supplementary material for: Effects of a Sleep Health Education Program for Children and Parents on Child Sleep Duration and Difficulties: A Stepped-Wedge Cluster Randomized Clinical Trial
Source: JAMA Netw Open. 2022 Jul 26;5(7):e2223692. doi: 10.1001/jamanetworkopen.2022.23692 (PMC9327577; doi:10.1001/jamanetworkopen.2022.23692)
Supplement: Supplement 1. — Trial Protocol and Statistical Analysis Plan [file jamanetwopen-e2223692-s001.pdf]

Trials structured Study Protocol template

Trials guidance: the numbers in curly brackets (e.g. {5a}) are SPIRIT item identifiers. **Please do not remove the numbers in curly brackets, or any heading that contains them**. The item identifiers are slightly out of sequence to make the document flow more easily but it is important that they remain in the document to allow electronic searches by SPIRIT item number.

If you are certain that an item does not apply, please state "n/a" and provide a short explanation. Leaving an item blank or stating “n/a” without an explanation will lead to your manuscript being returned before review.

NB: All text in green can be removed once you have finished creating your Study Protocol. Text in black is mandatory. Please also read the submission guidelines for Study Protocols prior to submitting to *Trials*: https://trialsjournal.biomedcentral.com/submission-guidelines/preparing-your-manuscript/study-protocoll

Title

Increasing Sleep Health Literacy: A Social Ecological Approach

Names protocol contributors

Karen Bonuck, PhD; Ronald Chervin MS, MD, Akilah Collins Anderson, MPH; Barbara True-Felt

Abstract

- **Background**: Inadequate and/or poor quality sleep in early childhood impairs social-emotional and cognitive function (via effects on the developing brain), and markedly increases obesity risk (via hormonal and endocrine effects). Short sleep duration, behavioral sleep problems and sleep-disordered breathing peak at 20%-50%, during the preschool years (ages 3-5). Healthy sleep habits increase sleep duration and prevent behavioral sleep problems. Awareness of sleep-disordered breathing symptoms leads to timely treatment for it. Despite ample data on sleep problems "…much less work has been done on effective strategies to promote sleep as a healthy behavior (CDC 2013)". This study's overarching goal is to empower families of preschool children with the knowledge and skills needed for healthy sleep, and to recognize a sleep problem. It builds on work in Head Start, an early childhood education (ECE) program for disadvantaged preschool children and their families: The team's Early Childhood Sleep Education Program (ECSEP™) educates Head Start teachers, children, and parents about healthy sleep in a way they can process and understand. In a randomized controlled trial, the children in the ECSEP group slept 30 minutes longer/night.
- **Methods**: The proposed study will implement a Social-Ecological web of multi-level interventions to reinforce the ECSEP, and to promote healthy sleep throughout ECE. Within Head Start, the team will create new delivery platforms (print & video, family

visits) that 'amplify' the ECSEP. Beyond Head Start, the team will educate communities, and partner with stakeholders on strategies designed to embed 'sleep health literacy' in ECE policy. This project will: 1) Adapt sleep education material into additional multi-media formats, and; apply the Health Care Institute model to train Head Start staff to mount interventions and collect data. 2) Enroll 540 parent-child dyads from 7 Head Start agencies in New York in a stepped wedge randomized controlled trial. Investigators will analyze trial effects on primary outcomes: a) child sleep duration, b) parent knowledge, attitudes, self-efficacy and behavior, and c) child sleep difficulties. 3) Assess the feasibility of screening and guidance for sleep problems (vs. sleep health) for a future efficacy study. Secondary outcomes are: classroom behaviors, policy change, and process data.

- **Discussion**: Poor sleep in early development has ramifications for years to come, perhaps through adulthood. Head Start serves low-income, mainly racial-ethnic minority families, in whom sleep health disparities are greatest-- but are modifiable.

Trial registration: NCT03556462

Keywords

Trials guidance: sleep health, children, sleep, Head Start

Administrative information

Note: the numbers in curly brackets in this protocol refer to SPIRIT checklist item numbers. The order of the items has been modified to group similar items (see http://www.equator-network.org/reporting-guidelines/spirit-2013-statement-defining-standard-protocol-items-for-clinical-trials/).

Title {1}	Stepped Wedge Cluster Randomized Controlled Trial of Parent and Child Sleep Health Education Interventions in Head Start]
Trial registration {2a and 2b}.	Clinical Trials.gov. NCT03556462.<br><br>Item 2b- na.
Protocol version {3}	Version 1. 5.16.2022]

Funding {4}	NICHD R01HD082129-01
Author details {5a}	KB, ACA (at time of study), CS: Einstein College of Medicine-Montefiore Medical Center<br>RC, BF: University of Michigan
Name and contact information for the trial sponsor {5b}	NICHD funded the study, which is complete.]
Role of sponsor {5c}	Study funding provided]

Introduction

Background and rationale {6a}

Young children need sufficient, healthy sleep for optimal cognitive, social-emotional, and physical development, and to reduce obesity risk. At preschool-age (3-5 years), 10-13 hours of sleep per day inclusive of naps, is recommended. Yet, prior to school-entry up to one-third of US children don't sleep this much Additionally, 25-30% of preschoolers experience difficulties falling and staying asleep, i.e., behavioral sleep problems (BSPs). Yet, optimal sleep hygiene practices at this age— a consistent bedtime and bedtime routine, falling asleep on one's own, limiting screen time before bed, etc.— are associated with fewer BSPs and longer sleep duration. Parent knowledge and attitudes about child sleep are significantly associated with the quality and quantity of young children's sleep. Generally, improved sleep hygiene and parent education are associated with positive effects on children's sleep and household routine, and no risks.

Objectives {7}

This was a primary prevention trial of sleep health interventions in Head Start. This RCT of multi-level interventions grounded in the social-ecological model examined the following outcomes at ~9- and 12-month follow-up: a) child sleep duration, b) child sleep difficulties, and c) parent knowledge, attitudes, beliefs, and self-efficacy (KASB) regarding children's sleep.

-SLEEP DURATION (CHILD): Hypothesized differences in sleep duration for Intervention vs. Control periods were: a) longer duration for Intervention periods (Primary Outcome: difference at FU3 [app. 9 month follow-up]), b) >30-minute difference at FU3, and c) a 15-minute difference in duration at FU4 (app. 12 months). Sleep duration was measured via parent-reported sleep logs.

-SLEEP DIFFICULTIES (CHILD): Hypothesized differences between Intervention vs. Control periods were: a) lower mean total scores, b) lower odds of scores >= 8/36 and c) fewer parents reporting a sleep problem in the 10th Tayside item (Yes/No). Surveys with at least 7 of 9 items scored were included in analysis. The Tayside Children's Sleep Habits Questionnaire was used to assess this outcome. (Secondary outcome)

-KNOWLEDGE-ATTITUDES-SELF EFFICACY- BELIEFS (PARENT): Hypothesized differences in parents'

KASB for Intervention vs. Control periods were: a) higher KASB total scores and b) higher scores for each domains. KASB scales with at least 80% non-missing responses were included in analysis. The KASB survey was developed to align with the main educational intervention, the Early Childhood Sleep Education Program. (Secondary outcome)

Trial design {8}

Stepped Wedge Cluster Randomized Controlled Trial. The 7 agencies were randomized: 4 agencies (and 12 sites that operate under them) and 3 agencies (and 11 sites that operate under them) were randomized to Wedge 1 and Wedge 2, respectively.

Methods: Participants, interventions and outcomes

Study setting {9}

7 Head Start agencies across New York State were randomized to implement interventions in either Fall 2018 or Winter/Spring 2019

Eligibility criteria {10}

From March thru September 2018 Head Start staff recruited a) English and/or Spanish speaking parents, b) of children 3 years of age on/about September 2018, c) who planned to remain at the site through the school year.

Who will take informed consent? {26a}

Head Start staff obtained informed consent

Additional consent provisions for collection and use of participant data and biological specimens {26b}

NA- the only study data were de-identified demographics from agencies and parent-completed surveys regarding their child's sleep and their knowledge, attitudes, self-efficacy and beliefs about child sleep.

Interventions

Explanation for the choice of comparators {6b}

NA- cross-over design

Intervention description {11a}

The study was implemented across the 2018-2019 school year. Head Start staff in each Wedge received half-day trainings one month prior to implementing the ECSEP's 1-hour Parent Workshop and 2-week classroom curriculum, and the Sleep Health Flipchart guided discussion. See Table 1:

Table 1: Intervention Descriptions: Content, Delivery and Materials

Early Childhood Sleep Education Program (ECSEP)™			
	Content:	***Delivery:***	***Materials:***
**Parent Workshop (ECSEP)**	• Science or sleep<br>• Sleep effects on child's brain & body<br>• Hours of sleep needed<br>• Bedtime routines (why, 'how to')	• Held at sites<br>• During parent meeting<br>• 1-week before class lessons	• 1-hour power-point<br>• Parents receive take-home guide
**Classroom Lessons (ECSEP)***	• Why children need sleep, bedtime routine steps, etc.<br>• Modalities: songs, storybooks, teddy bears to model bedtime routines	• By Head Start teachers<br>• Lessons: 8 sessions over 2 weeks, 40 min//day<br>• Small & large group activity	*Child take-homes*:<br>• Teddy Bear (2nd week)<br>• Book (read in class)<br>• Toothbrush & toothpaste<br>• Sticker chart &stickers<br>*Teacher tools*:<br>• Curriculum guide, lesson plans, 3 classroom bears, 10 enrichment lessons[b]
**Multimedia Materials Developed During Pre-Implementation**			
	Content:	***Delivery:***	***Materials:***
**Sleep Health Flipchart[a]** (Parent 1-to-1 with Head Start Staff)	• Reviews Parent Workshop content<br>Additional tailored content on:<br>• FAQs: night-waking, co-sleeping etc.<br>• Red flags: snoring, daytime fatigue	• At home or Head Start site<br>• During routine 1-to-1 visits<br>• Guides staff & parent dialogue<br>• Duration: 20-25 minutes	• Spiral-bound, desk-sized, full color<br>• 26 pages: 13 parent- and<br>• 13 staff-facing<br>• English and Spanish
**Sleep Health Brochure**	• Summarizes Flipchart content<br>• Includes: bedtime 'do's & don'ts<br>• Sleep Goals: parents can write 1-3	• Parent receives after Flipchart sessions	• Tri-fold brochure<br>• English and Spanish
**Bedtime Challenges Brochure***	• Strategies for: fighting bedtime, night-time fears, shared sleep space, etc.	• Parent receives after Flipchart sessions	• 1-page, double-sided<br>• English and Spanish
**Sleep Health Video***	• Professional production, content mirrors above materials	• Shared via agency social media, website, newsletter,	• 8-minute video distributed as .mp4<br>• English and Spanish

[a] One agency delivered the Sleep Health Flipchart in small groups immediately after the 1-hour Parent Workshop
[b] Supplemental lessons for future use; not implemented during trial

Criteria for discontinuing or modifying allocated interventions {11b}

NA- no changes were made.

Strategies to improve adherence to interventions {11c}

NA- There were no particular strategies to promote adherence to the classroom curriculum as children were present on site for the lessons. Regarding strategies to promote attendance at the 1-hour parent workshop, the event was held during times convenient for families, and some sites served refreshments.

Relevant concomitant care permitted or prohibited during the trial {11d}

NA- there was no other sleep education provided or prohibited.

Provisions for post-trial care {30}

NA- there was no follow-up beyond the 12 month (follow-up 4) survey data.

Outcomes {12}

Outcomes were parent-reported school-night sleep duration per sleep logs (primary: at 9 months; secondary: at 12 months), mild/moderate sleep difficulties per validated questionnaire, and KASB total and domain scores at 9 and 12 months (secondary)

Participant timeline {13}

Control | Intervention

	April-Sept KASB	Sept 2018	Nov 2018	Jan 2019	Mar 2019	Sept 2019
	Baseline	Baseline	Follow-Up Data 1	Follow-Up Data 2	Follow-Up Data 3	Follow-Up Data 4
Wedge 1		1-week before Wedge 1 Interventions	1-week after Wedge 1 Interventions	1-week before Wedge 2 Interventions	1-week after Wedge 2 Interventions	1 Year after Baseline
Wedge 2	KASB TAYSIDE	Log	Log KASB TAYSIDE	Log	Log KASB TAYSIDE	Log KASB TAYSIDE
	KASB TAYSIDE	Log	Log KASB TAYSIDE	Log	Log KASB TAYSIDE	Log KASB TAYSIDE

Figure 2

Sample size {14}

Mean school night sleep duration at FU3 (app. 9 months) was the primary outcome. Clinically significant effects are evident from an additional 30-35 minutes of nighttime sleep.[42,43] With 7 agencies, a sample of n=173 provides 90% power to detect a difference as small as 15 minutes between Intervention vs. Control phases (p<.05, 2-tail). For parent KASB, a secondary outcome, a sample of n=450 provides >97% power to detect a moderate effect size (Cohen's d = 0.3) for each of its named scales.

Recruitment {15}

There were no specific strategies to reach each agency's targeted recruitment goal.

Assignment of interventions: allocation

Sequence generation {16a}

Randomization was at the agency level. The 7 agencies were randomly assigned by a computer to implement interventions in Fall 2018 (Wedge 1) or Spring 2019 (Wedge 2)

Concealment mechanism {16b}

NA- this was infeasible as sites needed to prepare to implement the intervention in Fall 2018 or Spring 2019.

Implementation {16c}

As noted above, the study statistician generated the allocation of the 7 agencies to either Wedge 1 or Wedge 2.

Assignment of interventions: Blinding

Who will be blinded {17a}

NA- see above. Sites and families were cognizant of when the intervention(s) were offered.

Procedure for unblinding if needed {17b}

NA- see above..

Data collection and management

Plans for assessment and collection of outcomes {18a}

During these 2.5 years, the study team and partners developed and piloted intervention materials and built Head Start capacity to enroll participants, deliver interventions and collect study data. A kick-off retreat in March 2018, just prior to recruitment, was held to review logistics and materials. Researchers trained agency staff to enroll families and administer study instruments. About one month prior to each site's implementation of interventions, developers of the ECSEP™ (Sweet Dreamzzz, Inc., now part of Pajama Program) provided half-day trainings to staff delivering the Parent Workshop, Classroom Lessons and Sleep Health Flipchart .

Child Sleep Duration- This was measured from sleep log data for school nights (Sunday through Thursday). At recruitment, agency staff showed parents how to record their child's bed- and wake-times, defined as "lights out" and "when the child woke up to start the day," respectively. Parents then completed 7-day paper-and-pencil sleep logs at 5 points:1 week before/after implementation in Wedges 1 and 2, and at final 1-year follow-up (FU4).

Child Sleep Difficulties: This was assessed with the Tayside Children's Sleep Habits Questionnaire (Tayside), This valid and reliable 10-item tool assesses difficulties initiating and maintaining sleep in 1-5 year olds.[37,38] This low literacy (<= 6th grade) questionnaire is used in research.[39-41] For this RCT we reduced the recall period to 2 months (from 3), and edited language for clarity (e.g., double negatives). The first nine items were each scored from 1-4; a total score of >= 8/36 indicates mild/moderate difficulties.

Parent Knowledge/Attitude/Self-efficacy/Beliefs (KASB) Questionnaire: The KASB Questionnaire reflects content from the ECSEP Parent workshop regarding child sleep across the named domains. Parents rate agreement with 27 items on a 5-point scale for Knowledge (n=12); Attitudes (n=5); Self-efficacy (n=8) and Beliefs (n=2). One multiple-choice item asks how much sleep a preschooler requires.

Plans to promote participant retention and complete follow-up {18b}

Head Start staff see/interacted with parent-child dyads on a daily basis, and so were able to promote retention and follow-up.

Data management {19}

Head Start staff collected paper surveys and sleep logs. Researchers trained staff to enter data into site-specific REDCap secure databases. Paper surveys/logs were mailed back to the researchers who re-entered or corrected data as needed. Range checks were implemented. The original funding application identifies REDCap as the data storage systema and double-entry.

Confidentiality {27}

SPIRIT guidance: Data were collected from the parent participants- who completed surveys and logs themselves unless assistance was deemed of benefit- and entered by trained staff at the Head Start site. After data entry was complete, sites were no longer able to access the database.

Plans for collection, laboratory evaluation and storage of biological specimens for genetic or molecular analysis in this trial/future use {33}

NA- there were no such specimens in the study.

Statistical methods

Statistical methods for primary and secondary outcomes {20a}

STATISTICAL ANALYSIS PLAN

Summary statistics of continuous variables were calculated as means and standard deviations; frequency distributions (n and %) are shown for discrete variables. To analyze treatment effect we fitted multi-level generalized linear models with the study outcome variables as dependent variables, an indicator for post-intervention status, indicators for the study phase (follow-ups 1 through 4, baseline as reference category), and covariates to adjust for age, child sex, Hispanic ethnicity, and race. Random intercepts at the agency and participant levels were included. For continuous outcomes (sleep duration, questionnaire scale scores) the identity link and gaussian error family were used. The coefficient of the post-intervention indicator is the estimate of the average treatment effect and is presented along with its 95% confidence interval (CI). For the dichotomous outcomes (positive Tayside questionnaire, parent-reported sleep problem), the log link and logistic error family were used. In this model the treatment is the odds ratio (exponentiated coefficient) of the post-intervention indicator, presented with its 95% CI.

Interim analyses {21b}

NA- there were no analyses planned until after the 12-month follow-up.

Methods for additional analyses (e.g. subgroup analyses) {20b}

NA- there were no pre-planned subgroup analyses.

Methods in analysis to handle protocol non-adherence and any statistical methods to handle missing data {20c}

NA- there was no such protocol

Plans to give access to the full protocol, participant level-data and statistical code {31c}

The datasets generated during and/or analysed during the current study are available from the corresponding author on reasonable request

Oversight and monitoring

Composition of the coordinating centre and trial steering committee {5d}

NA- there was no coordinating centre or trial steering committee.

Composition of the data monitoring committee, its role and reporting structure {21a}

No data monitoring committee proposal was submitted to the NIH or required by the IRB given that this was a low-risk health education (behavioral) intervention

Adverse event reporting and harms {22}

NA- There were no plans to report adverse events as this was a low-risk behavioral intervention.

Frequency and plans for auditing trial conduct {23}

NA- Trial is completed

Plans for communicating important protocol amendments to relevant parties (e.g. trial participants, ethical committees) {25}

NA- there were no changes planned or made.

Dissemination plans {31a}

Each agency's results were transmitted to the agency's executive directors. Findings will, we anticipate, be published in JAMA Network Open, and disseminated (upon publication) through Einstein College of Medicine/Montefiore Medical Center social media.

Discussion

NA

Trial status

Trials guidance: Recruitment began May 18, 2019 and is now complete.

Abbreviations

Abbreviations are defined in the

text at first use.

Declarations

Trials guidance: The manuscript contains the following subheadings:

- Acknowledgements
- Authors' contributions
- Funding
- Availability of data and material
- Ethics approval and consent to participate
- Consent for publication
- Competing interests
- Authors’ information (optional)

Acknowledgements

NA- no other individuals contributed to the preparation of this manuscript

Authors’ contributions {31b}

SPIRIT guidance: [31b] -
KB- as PI, made substantial contributions to the conception **and** design of the work; **and** the acquisition, analysis, **and** interpretation of data; **and** drafted the work and substantively revised it.

ACA- made substantial contributions to the design of the work, acquisition of data; **and** substantively revised it.

RC -contributed to the conception and design of the work; and interpretation of data; **and** drafted the work or substantively revised it.

BF- contributed to the conception and design of the work; and interpretation of data; **and** drafted the work or substantively revised it.

CS- made substantial contributions to the conception **and** design of the work; **and** the analysis and interpretation of data; **and** drafted the work or substantively revised it.

All authors reviewed and approved the final version of the manuscript.
"

Funding {4}

This work was support be a grant from the National Institute of Child Health and Human Development: R01 HD082129.The funding body in the design of the study and collection, analysis, and interpretation of data and in writing the manuscript should be declared.

Availability of data and materials {29}

SPIRIT guidance: Einstein investigators have access to the final database.

Ethics approval and consent to participate {24}

SPIRIT guidance: Plans for seeking research ethics committee/institutional review board (REC/IRB) approval.

Trials guidance: Trials do not consider study protocols for studies without ethical approval. You will be required to provide a copy of the original ethical approval document and an English translation of this document as an additional file on submission, which will be checked against this declaration. The name of the ethics committee that approved the study and the committee's reference number (if applicable) should be declared. Details of authors' intentions to obtain consent to participate in the study from participants (or their parent or legal guardian in the case of children under 16) should be declared. "eg. ABC Ethical Review Board ABC123456. Written, informed consent to participate will be obtained from all participants"

Consent for publication {32}

SPIRIT guidance: Model consent form and other related documentation given to participants and authorised surrogates.

Trials guidance: Please do not include any baseline or pilot data in your study protocol. The Editorial Office will ask you to remove this if it is included. If you have included any details, images or videos relating to an individual person, written informed consent for the publication of these details must be obtained from that person (or their parent or legal guardian in the case of children under 18) and declared in this section. Please also state whether you will be willing to provide a model consent form on request. If this section does not apply, please state "Not applicable".

Competing interests {28}

SPIRIT guidance: Financial and other competing interests for principal investigators for the overall trial and each study site.

Trials guidance: All financial and non-financial competing interests must be declared in this section. See

our editorial policies for a full explanation of competing interests. If you are unsure whether you or any of your co-authors have a competing interest please contact the editorial office. Please use the authors initials to refer to each authors' competing interests in this section. If you do not have any competing interests, please state: "The authors declare that they have no competing interests" in this section.

Authors' information (optional)

n

References

See accompanying manuscript
