# Supplementary material for: Effects of a Sleep Health Education Program for Children and Parents on Child Sleep Duration and Difficulties: A Stepped-Wedge Cluster Randomized Clinical Trial
Source: JAMA Netw Open. 2022 Jul 26;5(7):e2223692. doi: 10.1001/jamanetworkopen.2022.23692 (PMC9327577; doi:10.1001/jamanetworkopen.2022.23692)
Supplement: Supplement 2. — eFigure. Stepped-Wedge Cluster Design eTable 1. Description of Sample at Baseline and End Point Follow-ups eTable 2. Parent Knowledge, Attitudes, Self-efficacy, and Behaviors (KASB): Regression Model Effects eTable 3. Additional Analyses: Intervention Effect on Sleep Duration [file jamanetwopen-e2223692-s002.pdf]

Supplementary Online Content

Bonuck K, Collins-Anderson A, Schechter CB, Felt BT, Chervin RD. Effects of a sleep health education program for children and parents on child sleep duration and difficulties: a stepped-wedge cluster randomized clinical trial. *JAMA Netw Open*. 2022;5(7):e2223692. doi:10.1001/jamanetworkopen.2022.23692

eFigure. Stepped-Wedge Cluster Design
eTable 1. Description of Sample at Baseline and End Point Follow-Ups
eTable 2. Parent Knowledge, Attitudes, Self-efficacy, and Behaviors (KASB): Regression Model Effects
eTable 3. Additional Analyses: Intervention Effect on Sleep Duration

This supplementary material has been provided by the authors to give readers additional information about their work.

eFigure. Stepped-Wedge Cluster Design

Control | Intervention

	April-Sept KASB	Sept 2018	Nov 2018	Jan 2019	Mar 2019	Sept 2019
	Baseline	Baseline	Follow-Up Data 1	Follow-Up Data 2	Follow-Up Data 3	Follow-Up Data 4
		1-week before Wedge 1 Interventions	1-week after Wedge 1 Interventions	1-week before Wedge 2 Interventions	1-week after Wedge 2 Interventions	1 Year after Baseline
**Wedge 1**	KASB TAYSIDE (Control)	Log (Control)	Log KASB TAYSIDE (Intervention)	Log (Intervention)	Log KASB TAYSIDE (Intervention)	Log KASB TAYSIDE (Intervention)
**Wedge 2**	KASB TAYSIDE (Control)	Log (Control)	Log KASB TAYSIDE (Control)	Log (Intervention)	Log KASB TAYSIDE (Intervention)	Log KASB TAYSIDE (Intervention)

© 2022 Bonuck K et al. *JAMA Network Open*.

eTable 1. Description of Sample at Baseline and End Point Follow-Ups, n (%) or mean (sd)

	**Baseline N=519**	**Follow-Up 3 N= 327**	**Follow-Up 4 N= 282**
Child Age: Years at Baseline	2.7 (0.1)	2.8 (0.1)	2.7 (0.1)
Child Gender)			
Girls	264 (50.9%)	177 (54.1%)	145 (51.4%)
Boys	239 (46.1%)	149 (45.6%)	137 (48.6%)
Language			
Spanish	196 (37.8%)	136 (41.6%)	107 (37.9%)
English	319 (61.5%)	190 (58.1%)	173 (61.3%)
Other	2 (0.4%)	0 (0%)	2 (0.7%)
IFSP/IEP [a]: Yes	31 (9.8%)	25 (13.5%)	20 (10.9%)
Race (not mutually exclusive)			
American Indian/Alaskan Native	5 (0.9%)	4 (1.2%)	4 (1.4%)
Asian American/Pacific Islander	17 (3.2%)	13 (3.9%)	7 (2.4%)
Black	57 (10.8%)	43 (12.9%)	24 (8.3%)
White	199 (37.8%)	103 (30.9%)	136 (47.2%)
Other	63 (12.0%)	53 (15.9%)	39 (13.5%)
Ethnicity: Hispanic	202 (44.2%)	160 (52.5%)	106 (41.6%)
Child BMI: 2018	16.9 (0.6)	16.9 (0.5)	16.6 (0.6)
Child BMI: 2019	16.4 (0.3)	16.6 (0.4)	16.4 (0.4)

[a] Individualized Family Service Plan, Individualized Education Plan

© 2022 Bonuck K et al. *JAMA Network Open*.

eTable2. Parent Knowledge, Attitudes, Self-efficacy, and Behaviors (KASB): Regression Model Effects (higher score= better)

	Effect	Items: mean # 'Correct'	Lower 95% CI	Upper 95% CI	p
**Total KASB**	Post-Intervention	0.81	-1.29	2.90	0.45
Age	Years	1.27	-1.11	3.64	
Gender	Female	0.18	-1.14	1.49	
Ethnicity	Hispanic	-2.49	-4.78	-0.20	
Race	Black	-1.89	-4.84	1.06	
	AI/AN	-1.13	-7.03	4.78	
	Asian PI	-2.71	-6.88	1.46	
	White	-2.27	-4.90	0.36	
	Other	-0.91	-3.38	1.55	
Knowledge	Post-Intervention	1.13	0.13	2.12	0.03
Age	Years	-0.01	-1.18	1.15	
Gender	Female	-0.18	-0.83	0.46	
Ethnicity	Hispanic	-2.25	-3.37	-1.13	
Race	Black	-0.13	-1.57	1.31	
	AI/AN	1.40	-1.50	4.31	
	Asian PI	-1.27	-3.29	0.75	
	White	-0.56	-1.78	0.67	
	Other	0.72	-0.42	1.86	
Attitudes	Post-Intervention	0.16	-0.46	0.77	0.62
Age	Years	0.24	-0.57	1.06	
Gender	Female	0.32	-0.13	0.78	
Ethnicity	Hispanic	-0.16	-0.94	0.63	
Race	Black	-0.97	-1.98	0.04	
	AI/AN	-0.97	-3.05	1.10	
	Asian PI	0.03	-1.39	1.44	
	White	-0.65	-1.46	0.16	
	Other	-0.44	-1.18	0.30	
Self-Efficacy	Post-Intervention	-0.13	-1.02	0.76	0.78
Age	Years	0.78	-0.33	1.89	
Gender	Female	0.14	-0.47	0.76	
Ethnicity	Hispanic	-0.13	-1.20	0.94	
Race	Black	-0.53	-1.91	0.85	
	AI/AN	-1.51	-4.32	1.29	
	Asian PI	-0.87	-2.82	1.07	
	White	-1.20	-2.36	-0.04	
	Other	-1.15	-2.23	-0.07	
Beliefs	Post-Intervention	-0.20	-0.56	0.16	0.28
Age	Years	0.16	-0.20	0.51	
Gender	Female	-0.04	-0.24	0.16	
Ethnicity	Hispanic	-0.28	-0.62	0.06	
Race	Black	-0.18	-0.63	0.26	
	AI/AN	-0.04	-0.90	0.82	
	Asian PI	-0.40	-1.03	0.24	
	White	-0.17	-0.63	0.29	
	Other	-0.05	-0.49	0.40	

© 2022 Bonuck K et al. *JAMA Network Open*.

eTable3. Additional Analyses: Intervention Effect on Sleep Duration

Covariates Added to Model:	Effect Estimate (minutes)	Lower 95% CI	Upper 95% CI	p
Site Level:				
Site (random effect)	6	-1.8	14.4	0.13
Site (indicators)	7.8	0	16.2	0.06
Urban vs Rural indicator	5.4	-2.4	13.2	0.17
Participant Level:				
Language	5.4	-2.4	13.8	0.17
Tayside: Baseline total score	6	-2.4	14.4	0.15
Tayside: Concurrent total score	5.4	-4.2	15.6	0.27
Tayside: Sleep Difficulties (parent report)	5.4	-3	13.2	0.20
Tayside: Consistency of parent report with total score	6	-2.4	13.8	0.16

© 2022 Bonuck K et al. *JAMA Network Open*.
