# Supplementary material for: Effects of a Sleep Health Education Program for Children and Parents on Child Sleep Duration and Difficulties: A Stepped-Wedge Cluster Randomized Clinical Trial
Source: JAMA Netw Open. 2022 Jul 26;5(7):e2223692. doi: 10.1001/jamanetworkopen.2022.23692 (PMC9327577; doi:10.1001/jamanetworkopen.2022.23692)
Supplement: Supplement 3. — Data Sharing Statement [file jamanetwopen-e2223692-s003.pdf]

Data Sharing Statement

Bonuck. Effects of a Sleep Health Education Program for Children and Parents on Child Sleep Duration and Difficulties. *JAMA Netw Open*. Published July 26, 2022. doi:10.1001/jamanetworkopen.2022.23692

Data
Data available: No

Additional Information
Explanation for why data not available: Upon request
